# Autophagy impairment by African swine fever virus

**DOI:** 10.1099/jgv.0.001637

**Published:** 2021-08-18

**Authors:** Gareth L. Shimmon, Joshua Y. K. Hui, Thomas E. Wileman, Christopher L. Netherton

**Affiliations:** ^1^​ The Pirbright Institute, Ash Road, Pirbright, Woking, Surrey GU24 0NF, UK; ^2^​ Biomedical Research Centre, University of East Anglia, Norwich Research Park, Norwich NR4 7TJ, UK; ^3^​ Quadram Institute, Norwich Research Park, Norwich, Norfolk, NR4 7UQ, UK

**Keywords:** African swine fever, DNA virus, host-pathogen interactions, immune evasion, transcription

## Abstract

African swine fever is a devastating disease of domestic swine and wild boar caused by a large double-stranded DNA virus that encodes for more than 150 open reading frames. There is no licensed vaccine for the disease and the most promising current candidates are modified live viruses that have been attenuated by deletion of virulence factors. Like many viruses African swine fever virus significantly alters the host cell machinery to benefit its replication and viral genes that modify host pathways represent promising targets for development of gene deleted vaccines. Autophagy is an important cellular pathway that is involved in cellular homeostasis, innate and adaptive immunity and therefore is manipulated by a number of different viruses. Autophagy is regulated by a complex protein cascade and here we show that African swine fever virus can block formation of autophagosomes, a critical functional step of the autophagy pathway through at least two different mechanisms. Interestingly this does not require the *A179L* gene that has been shown to interact with Beclin-1, an important autophagy regulator.

## Introduction

African swine fever virus (ASFV) causes an invariably fatal haemorrhagic disease of domestic pigs and wild boar that is now widely distributed across four of the world’s continents. Efforts to control the disease have been frustrated in part by the lack of an effective vaccine. Live attenuated viruses represent the mostly promising route to a commercial vaccine in the short term and modified live viruses produced by targeted gene deletion have a superior safety profile when compared to attenuated viruses generated by passage through tissue culture. Identifying genes capable of manipulating host cell responses has proven a fruitful way of identifying potential virulence factors [1–3] and gene deleted viruses based on these results have showed significant promise as vaccine candidates [4–9]. Recombinant virus lacking genes encoding for proteins that modulate lymphocyte proliferation [8, 10], block stress responses [11] or support virus replication [12] have proven effective at protecting animals from virulent challenge, however these strategies do not always translate to different ASFV genotypes [9, 10, 13, 14]. Gene deletions focused on a locus that modulates the host interferon response [1, 15] and encodes multiple multigene family 360 (MGF360) and MGF505 genes attenuates three different ASFV genotypes [4, 6, 9, 16].

Autophagy is a constitutive cellular process of bulk degradation that leads to elimination and recycling of cytosolic cell constituents by delivery to mammalian lysosomes or plant and yeast vacuoles [17]. To date, three distinct types of autophagy have been described, microautophagy, chaperone-mediated autophagy, and macroautophagy and in this text when we refer to ‘autophagy’ we refer to the process of macroautophagy which is centred on the incorporation of cargo into specialised vesicular organelles called autophagosomes. These migrate to and fuse with lysosomes which then breakdown cargo, which can include damaged organelles, long-lived proteins, and even invasive pathogens. Autophagy is regulated by a complex protein cascade that includes the mammalian target of rapamycin (mTOR) complex (mTORC), which acts on the ULK complex [18, 19], which in turns acts on the vacuolar protein sorting 34 (Vps34) lipid kinase complex [20] that includes a Bcl-2 like protein called Beclin-1 [21]. Two downstream ubiquitin like conjugation systems causes the covalent attachment of Atg12 to Atg5 to form the Atg16 complex, which then drives the lipidation of the Atg8 family of proteins, including LC3, which are major structural proteins of the nascent autophagosome. mTOR itself is regulated by the PI3K/Akt signalling cascade [22] and elevated levels of phosphorylated 4E-BP1 have been detected in Vero cells infected with a tissue cultured adapted strain of ASFV [23] suggesting that ASFV may also activate mTOR.

Autophagy acts as an intrinsic defence mechanism by degrading cytosolic or vacuole-containing microbes [24]. TLR4 activation can lead to release of Beclin-1 from Bcl-2 via binding to TLR adaptor proteins which initiate an autophagy response [25]. Additionally, evidence suggests that considerable cross-talk exists between autophagy and the interferon response [26]. For example, expression of RNA-dependent eIF2α protein kinase (PKR), an important interferon-stimulated gene (ISG) product is essential for autophagic degradation of HSV-1 [27]. Collectively, studies such as these demonstrate an integral role of autophagy in innate immunity. Cytosolic proteins have been shown to be constitutively delivered to multi-vesicular MHC class II-loading compartments via fusion with autophagosomes. In this way, targeting of the influenza matrix protein 1 to autophagosomes led to strongly enhanced MHC class II presentation to CD4+ T cell clones [28]. Autophagy can also influence MHC class I presentation and enhance the presentation of viral antigens during HSV-1 infection [29].

With such an important role in the host response to infection, it is unsurprising that autophagy is modulated by viruses. Herpesviruses are targeted for degradation by autophagy (a process referred to as xenophagy) and the HSV-1 Us11 protein has been shown to inhibit PKR-mediated induction of autophagy [30]. The ICP34.5 protein of HSV-1 contains a Beclin-1 interacting domain that binds and inhibits the Beclin-1’s ability to activate autophagy [31]. Beclin-1 is a common target for virus modulation and the ASFV A179L gene encodes for a Beclin-1 binding protein that has a high degree of sequence homology to the proto-oncogene Bcl-2 [32–34]. Beclin-1 denotes an important point of convergence between the apoptosis and autophagy pathways perhaps making it a particularly useful target for inhibition by viruses [35]. ASFV seems to exploit this cross-over as exogenous expression of A179L leads to the inhibition of both apoptosis and the formation of autophagosomes [33, 36, 37].

Modifying the ability of viruses to control the autophagy response can significantly alter the outcome of infection. Infection of mice with the recombinant HSV-1 mutant virus lacking the Beclin-1 binding domain of ICP34.5 resulted in reduced mortality compared to wild-type virus [31], demonstrating the potential for exploiting autophagy as a means of viral attenuation. A separate study using this virus showed that infection resulted in a significantly stronger CD4+ T cell response to recall antigen [38] and the cellular immune response plays an important role in protection against ASFV [39, 40]. Therefore, we reasoned that modulation of autophagy may play an important part in ASFV replication biology and could provide a target for rational design of vaccines. For example, the immunogenicity of vaccine strain viruses could be enhanced by limiting the ability of the virus to modulate autophagy which may lead to increased antigen presentation and a greater T-cell response.

## Methods

### Cells and viruses

Vero African green monkey kidney cells were cultured in DMEM supplemented with 10 % FCS, 25 mM HEPES and 100 U ml^−1^ streptomycin and 100 U ml^−1^ penicillin. The Vero adapted Badajoz 1971 strain (Ba71v) and Ba71vΔA179L have been described previously and were grown on Vero cells [41, 42]. The titre determined by endpoint dilution on Vero cells using expression of p30 as a marker for infection. Virus titrations were stained as for confocal microscopy below using anti-p30 clone C18, read using an inverted microscope and the TICD50 determined using the Spearman-Kärber method [43]. For infections, Vero cells were seeded at 2.5×10^4^ cells cm^−2^ and the following day virus was diluted in DMEM supplemented with 2 % FCS and incubated on cells for 1 h at 37 °C. The inoculum was removed, the cells washed and replaced with fresh 2 %-DMEM – this constituted the 0 h post-infection (hpi) time point.

### SDS-PAGE and immunoblotting

Proteins from cell lysates were resolved by SDS-PAGE (sodium dodecyl sulphate polyacrylamide gel electrophoresis) in NuPAGE MES running buffer (ThermoFisher Scientific) with the exception of gels for LC3 analysis that were run in NuPAGE MOPS running buffer (ThermoFisher Scientific). Proteins were transferred onto PVDF membranes for 1 h in transfer buffer (25 mM Trizma, 190 mM glycine, 20 % methanol), blocked in buffer consisting of TBS with 0.2 % (v/v) Tween (TBS-T) and 5 % (w/v) milk powder for 1 h, prior to overnight incubation with primary antibody diluted in blocking buffer. A list of antibodies used for both immunoblotting and confocal microscopy are provided in the supplemental data that accompanies this manuscript. Membranes were washed three times in TBS-T, prior to incubation with appropriate HRP-conjugated secondary antibodies, which had been diluted in blocking buffer, for 1 h. Membranes were washed a further three times in TBS-T prior to detection of chemiluminescence by incubating the membrane with Pierce ECL Western blotting substrate (ThermoFisher Scientific) for 3 min. Membranes were then imaged using a G:Box Chemi system (Syngene). The Gel Analysis function of ImageJ was used to calculate the relative density of loading controls and proteins of interest and the relative normalised density of proteins of interest derived by dividing the relative density of the protein of interest by that of the loading control.

### Confocal microscopy

Cells were fixed in 4 % PFA for 30 min followed by permeabilisation using methanol and then washed three times with PBS before incubating for 1 h in block buffer consisting of 10 % (v/v) TBS, 0.2 % (v/v) NaN3 and 0.2 % (v/v) fish skin gelatin (Sigma). Coverslips were then incubated for a further hour in primary antibody diluted in block buffer and then washed three times for 5 min in PBS to remove excess antibody. Next, coverslips were incubated for 1 h with an appropriate secondary antibody diluted in block buffer and then washed three times in PBS (-) before incubation with 1 µg ml^−1^ DAPI nuclear stain (4′, 6-diamidino-2-phenylindole, dihydrochloride, Sigma) in H_2_O for 10 min. Finally, coverslips were washed twice in ddH_2_O and then mounted in Vectashield (Vector Laboratories) on glass slides, and sealed using nail varnish. Cells were visualised using a Leica confocal laser scanning microscope, and data analysed using LAS X (Leica Confocal Software) and the ‘Cells’ analysis package of Imaris (Oxford Instruments Group, version 9.2.1) using the ‘Cells’ analysis function. Cells were detected using cytoplasmic LC3 staining or p30 labelling of protein expression following infection or transduction. Detection thresholds were adjusted to cover each cell to the cell boundary. The software was set to detect puncta of approximately 0.5 µm in diameter and cells were visually inspected to ensure that the software had accurately identified visible puncta. Analysis was carried out for at least 30 cells per experimental condition.

### Statistics

Statistical analysis of data was performed with Graphpad (Prism, version 8.2).

## Results

**Fig. 1.**
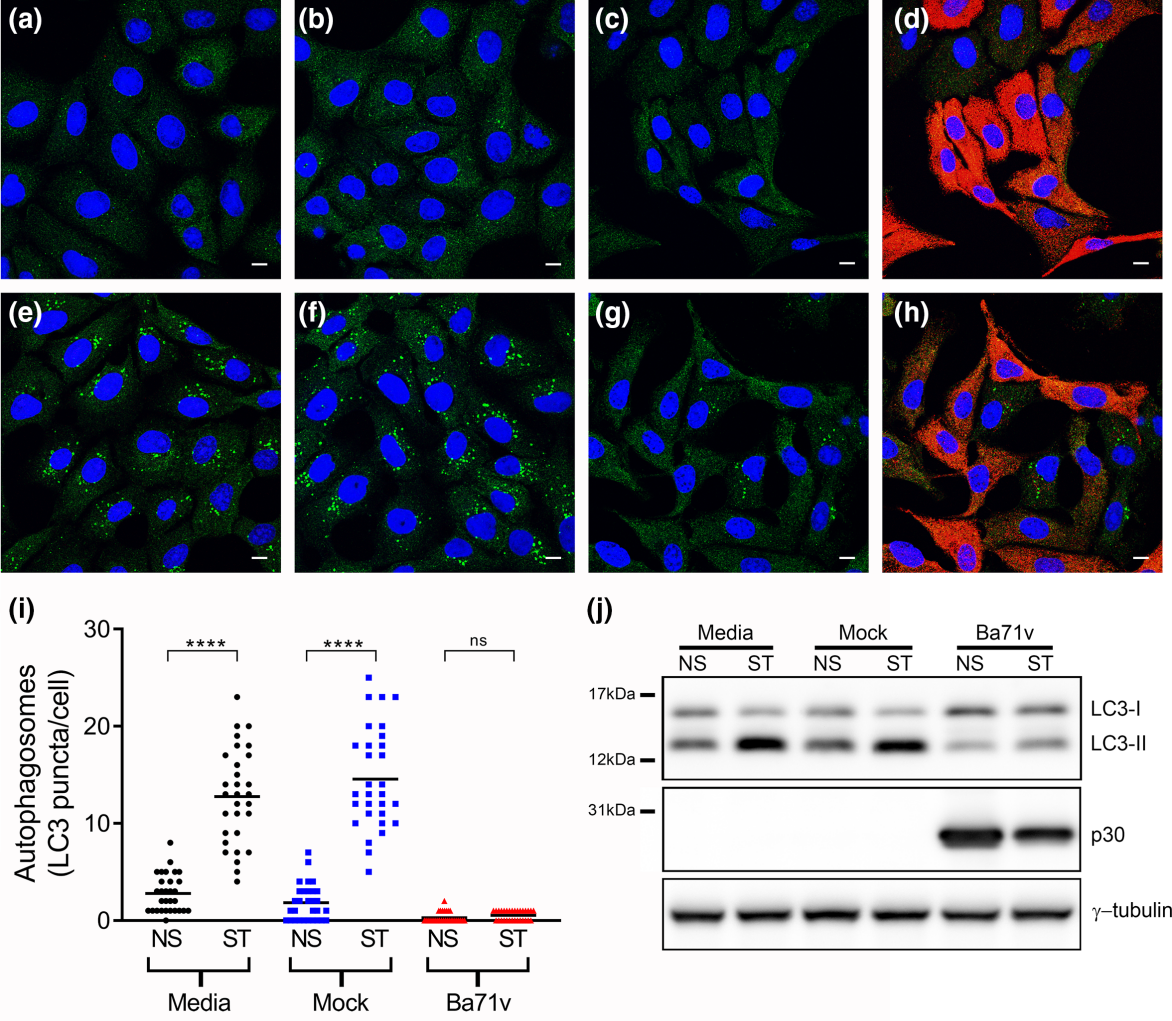
ASFV blocks starvation induced formation of autophagosomes. Vero cells were incubated with media alone (**a, e**), mock inoculum (**b, f**) or Ba71V (**c, d, g, h**) for 1 h. Inocula were removed and cells were incubated for a total of 4 h during which cells were either incubated in media (**a–d**) starved in EBSS for the final 2 h (**e–h**). Cells were then processed for, and analysed by, confocal microscopy (Panels a–i) or immunoblotting (Panel j). (**a–h**) Confocal images of cells labelled for LC3 (green), nuclei (blue) and p30 (red). Panels c and g shows the same infected cells as Panel d and h respectively, but with the red channel removed to allow for clearer observation of LC3 staining. Scale bars represent 10 µm. (**i**) The number of LC3 puncta per cell for 30 individual cells per indicated experimental condition was quantified by Imaris analysis of confocal images (NS - complete cell media, ST - starved in EBSS). Centre lines show the medians and differences between the indicated medians were tested using a Kruskal-Wallis test. Asterisks represent significant differences in value between NS and ST conditions ****=*P* value of <0.0001). (**j**) Immunoblots were probed with anti-LC3, anti-p30 and γ-tubulin antibodies. The positions of molecular mass markers are indicated to the left of the gels.

The effects of ASFV on autophagy 4 h post-infection (hpi) are shown in Fig. 1. Induction of autophagy was assessed by counting autophagosomes in cells starved in EBSS in the presence or absence of virus. Fig. 1(a, b) show that starvation induced autophagosomes labelled with LC3 in uninfected cells and mock infected cells, but numbers of LC3 puncta were reduced in cells infected with ASFV (Fig. 1c) This was quantified by counting puncta in cells counterstained for viral protein p30 (Fig. 1d, e). Starvation of control or mock infected cells in EBSS generated between 10 and 15 puncta per cell, but it was not possible to detect LC3 puncta in infected cells identified by immunostaining for p30. Autophagy can also be assessed by following the conversion of LCI to LC3II during the conjugation of LC3 to phosphatidylethanolamine (PE) in the autophagosome membrane. Western blot (Fig. 1f) shows that the levels of the faster migrating LC3II increased after starvation but LC3II levels were reduced in infected cells. Taken together with the confocal microscopy data this suggested that ASFV blocked starvation-induced formation of autophagosomes in Vero cells. Significant changes in autophagosome numbers over the course of sixteen hours were not observed (Fig. S1), consistent with analysis of levels of LC3II by immunoblotting [33]. Furthermore, ASFV was able to block the formation of starvation induced autophagosomes at all of the time points tested (Fig. S1). The ability of virulent field isolates to inhibit autophagy in porcine macrophages, which are the natural host cell of ASFV, was also tested. The high auto-fluorescence of macrophages made it difficult to identify LC3 puncta by microscopy. Autophagosome formation was therefore monitored by immunoblotting for LC3. Similar to what was observed in Vero cells, induction of autophagy in macrophages infected with the virulent OUR T88/1 ASFV isolate by Torin2 did not increase levels of LC3-II (Fig. S2) suggesting that field isolates of ASFV could inhibit autophagosome formation in primary macrophages.

**Fig. 2.**
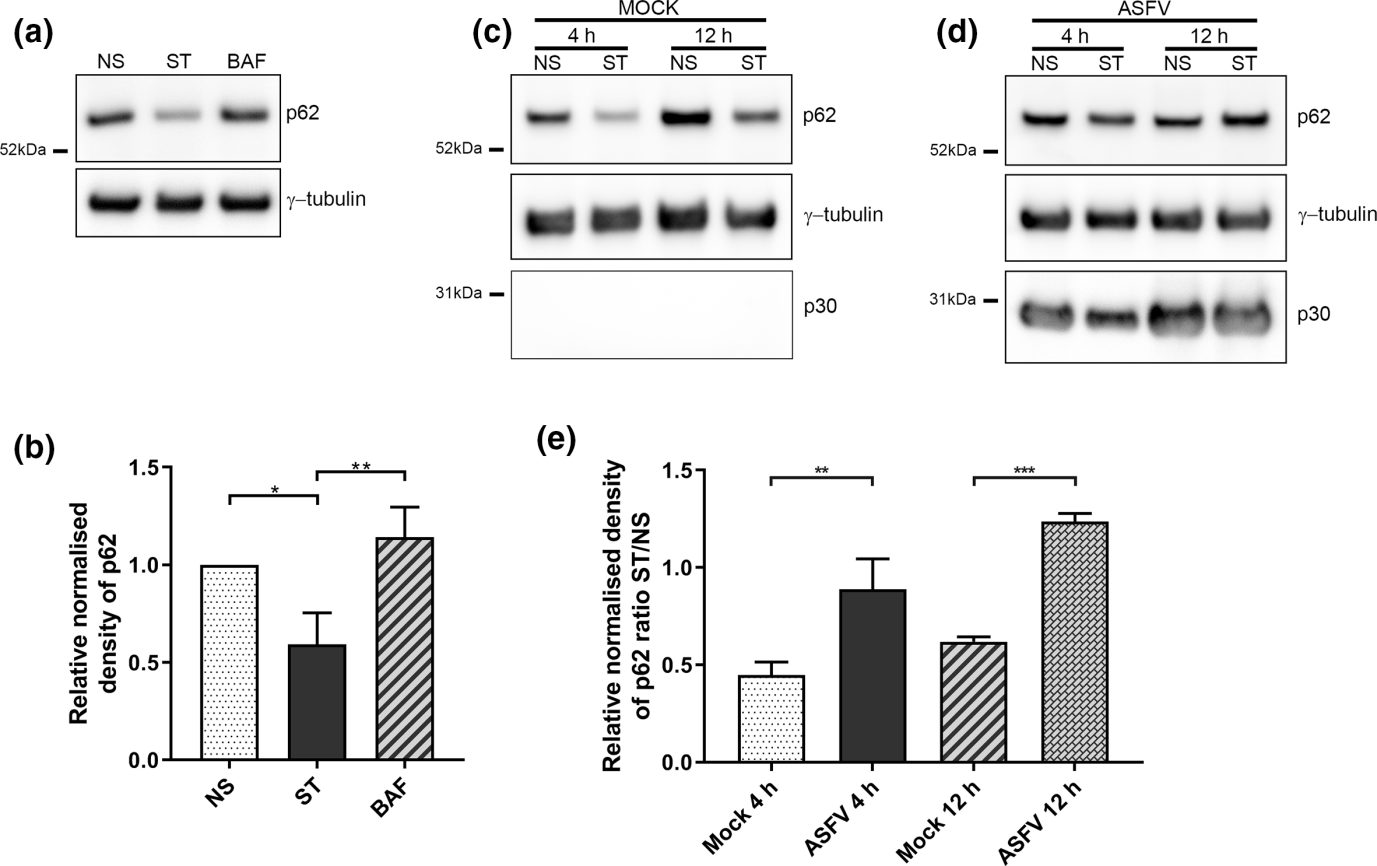
ASFV inhibits starvation-induced degradation of p62. (a) Vero cells were either incubated in complete cell media (NS), starved in EBSS (ST) or starved in EBSS in the presence of 100 nM bafilomycin A1 (BAF) for 1 h. Cells lysates were processed for immunoblot and probed with anti-p62 and γ-tubulin. (**b**) The relative densities of the p62 bands were calculated by comparison to that of the non-starved sample before being normalised to the values of γ-tubulin. (**c, d**) Vero cells were incubated with mock inoculum (Panel a) or Ba71V (MOI 5) (Panel b) for 1 h and then incubated for a total of 4 h or 12 h during which cells were either incubated in complete cell media (NS) or starved in EBSS (ST) for the final 2 h. Cells were lysed and immunoblots probed with anti-p62, anti-p30 and γ-tubulin antibodies. (**e**) Relative densities of the p62 bands were calculated by comparison to those of the 4 hpi non-starved sample before normalising the values to those of γ-tubulin. This was carried out separately on the mock infected and ASFV infected samples. Finally, the ratio of starved (ST) to non-starved (NS) values were calculated for mock infected and ASFV infected samples at 4 hpi and 12 hpi. (a, c, d) The positions of molecular mass markers are indicated to the left of the gels. Densitometry data represents the mean of three experiments and error bars indicate SD. Statistical analysis was carried out using one-way analysis of variance with Tukey multiple comparisons test. Asterisks represent a significant difference in value between the indicated conditions (*=*P* value of <0.05, **=*P* value of <0.01, ***=*P* value of <0.001).

The autophagy cargo protein, SQSTM1/p62 is degraded after the fusion of autophagosomes with lysosomes. This allows a fall in levels of p62 to serve as a useful index of autophagic degradation. Western blots (Fig. 2a, b) show that starvation of Vero cells for 1 h reduced steady state levels of p62 by approximately one half, and this was blocked by addition of the vacuolar H+ATPase inhibitor bafilomycin A1, that prevents fusion between autophagosomes and lysosomes [44]. This drop in p62 was seen in cells mock infected for 4 or 12 h prior to starvation, but the loss of p62 was not seen in when cells infected with ASFV were starved in EBSS (Fig. 2d, e)

Beclin-1 is part of the Vps34 lipid kinase complex upstream of the Atg5-Atg12 and Atg8/LC3 conjugation systems. Beclin-1 is held in an inactive complex with Bcl2 and dissociation of Beclin 1 from Bcl2 is required for autophagy induction [45]. The Bcl-2 homologue A179L encoded by ASFV interacts with Beclin-1 [33, 37, 46] and can prevent starvation induced formation of autophagosomes in transduced cells [37]. The possibility that A179L provided the inhibition of autophagy observed in Figs 1 and 2 was tested by assessing autophagy in starved cells infected with a viral strain carrying a deletion in A179L (Ba71vΔA179L). Figs S1 and S3 shows that autophagosome formation was inhibited following infection with Ba71vΔA179L suggesting that A179L is not necessary for viral inhibition of autophagy. A179L binds to peptides spanning the BH3 motifs of cellular Bcl2 proteins such as Bid, Bim, and Puma with between 50 to 300 times greater affinity than to peptides derived from Beclin-1 [46], therefore A179L may preferentially interact with other Bcl2 proteins in infected cells rather than Beclin-1. The apparent absence of a functional role for A179L in ASFV inhibition of autophagosome formation and the lack of other candidate ASFV encoded Bcl-2 homologues led us to investigate whether inhibition of autophagy by ASFV occurred earlier in the autophagy signalling cascade.

**Fig. 3.**
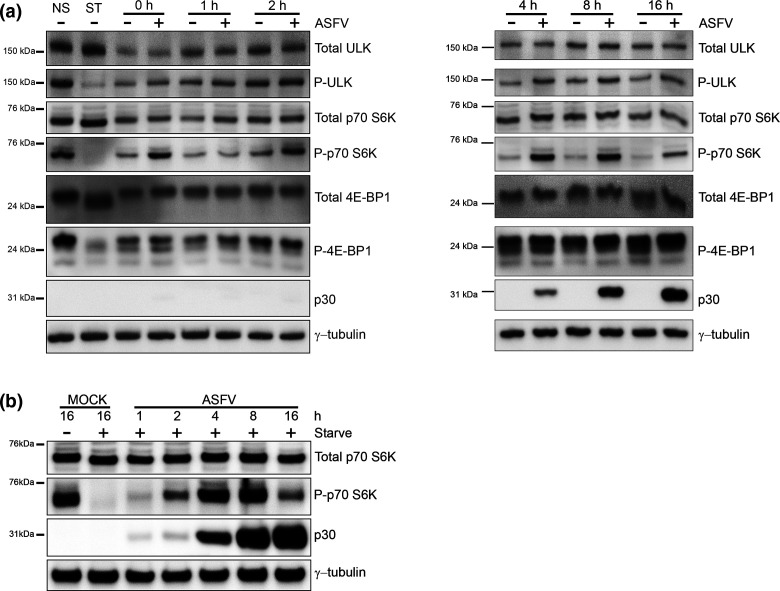
ASFV activates mTORC1 early during infection. (a) Vero cells were either mock infected or infected with Ba71V (MOI 5) for 1 h before residual virus was washed off and the 0 h time point was harvested. The remaining cells were incubated in 2 % media and harvested at multiple time points over a 16 h time course of infection. Control cells were either incubated in complete cell media (NS) or starved (ST) in EBSS for 3 h to induce inactivation of mTORC1. (**b**) Vero cells were infected with Ba71V (MOI 5) and harvested at multiple time points over a 16 h time course of infection. Prior to harvest, cells were starved for 1 h in EBSS. Separately, cells were mock infected for a total of 16 h and either incubated in complete cell media or starved for 1 h in EBSS prior to harvest. (a, b) Cells were lysed, processed for immunoblot and probed with the indicated antibodies. The time post-infection in hours (hpi) are indicated at the top of the gel, the positions of molecular mass markers are indicated to the left.

mTORC1 is activated in nutrient rich conditions and inhibits autophagy [47] by phosphorylating ULK1 to supresses the kinase activity of the ULK1 complex. At the same time mTORC1 phosphorylates p70-S6K and 4E-BP1 to increase translation of mRNA. The effect of ASFV on phosphorylation of ULK1 (P-ULK1), p70-S6K (P-p70-S6K) and 4E-BP1 (P-4E-BP1) in cells incubated in nutrient media or starved in EBSS is shown in Fig. 3(a). As expected, starvation of uninfected cells decreased phosphorylation of ULK1, p70 S6K and 4E-BP1 indicating inhibition of mTORC1. Levels of p-ULK1 remained constant during the first 4 hpi of infection and then rose slightly compared to mock infected cells up to 16 hpi suggesting up regulation of mTORC1 by ASFV. Interestingly, P-p70-S6K was elevated after 1 h preincubation with virus (0 hpi). Levels of P-p70-S6K then fell transiently at 1 hpi, but increased dramatically between 4 to 16 hpi. A similar, but less pronounced effect was seen with P-4E-BP1. Analysis of total protein levels of ULK1, p70-S6K and 4E-BP1 as well as γ-tubulin showed this was not due to changes in the steady state levels of the individual proteins. A similar pattern of phosphorylation of mTORC1 substrates was seen in macrophages infected with a virulent field strain (Fig. S4). The results suggested that ASFV inhibited autophagy by activating mTORC1 in cells in nutrient media. The activation of mTORC1 by ASFV was tested further by analysing p70-S6K in starved cells (Fig. 3b). P-p70-S6K was absent from mock infected cells, however levels of P-p70-S6K increased during ASFV infection peaking between 4 hpi and 8 hpi, before decreasing again at 16 hpi. This data suggested that activation of mTORC1 allows ASFV to circumvent activation of autophagy during starvation.

**Fig. 4.**
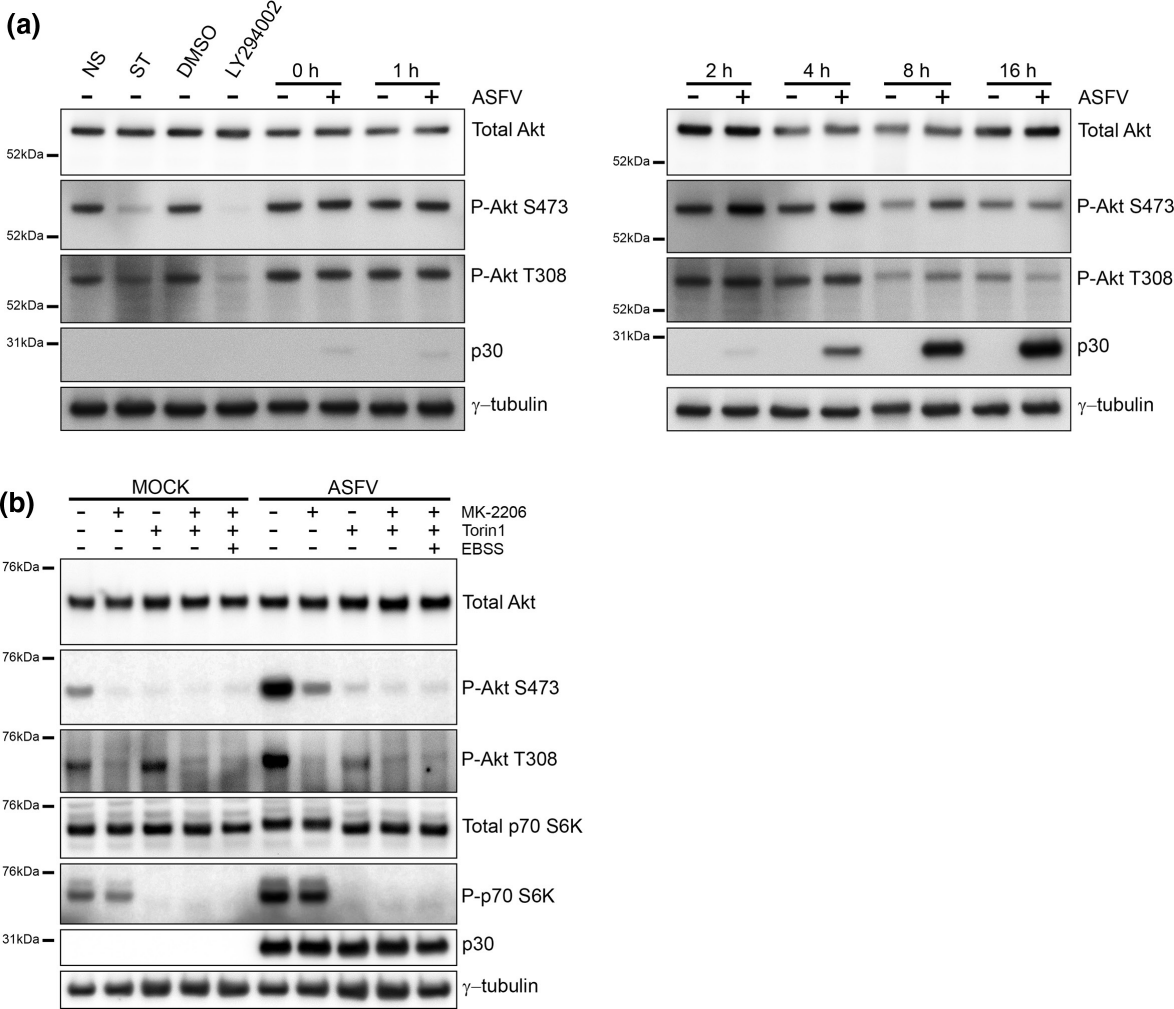
ASFV targets AKT. (a) Vero cells were either mock infected or infected with Ba71V (MOI 5) for 1 h before residual virus was washed off and the 0 h time point was harvested. The remaining infected cells were incubated in 2 % media and harvested at multiple time points over a 16 h time course of infection. Control cells were either non-starved (NS) in complete cell media, starved (ST) in EBSS, incubated in media containing DMSO or incubated in media containing 50 nM LY294002 for 3 h. (**b**) Vero cells were incubated with mock inoculum (mock infected) or Ba71V (MOI 5) for 1 h. Inocula were removed and cells were incubated for a total of 4 h. During the last 2 h, cells were incubated in regular 2 % media or in 2 % media containing 200 nM Torin1 or 5 µM MK-2206 or a combination of both drugs. Separately, cells were starved in EBSS in the presence of both drugs. (a, b) Cells were then lysed, processed for immunoblot and probed with antibodies against the indicated proteins. The positions of molecular mass markers are indicated to the left of the gels.

The activity of mTORC1 is increased by Akt either directly via Akt-mediated phosphorylation and suppression of TSC2, or via inhibition of AMPK [48]. Activation of Akt during ASFV infection was assessed by Western blot of phosphorylated T308 and S473 residues on Akt [49] (Fig. 4a). Starvation, or inhibition of Akt signalling by the PI3K inhibitor LY294002, led to a reduction in P-Akt T308, and a near absence of P-Akt S473. ASFV had little effect on phosphorylation of Akt T308, even so ASFV appeared to activate Akt early during infection indicated by raised levels of p-Akt S473 at 2 hpi, which reached a maximum at 4 and 8 hpi and S473 Akt remained phosphorylated throughout the ASFV replication cycle. This suggested that activation Akt may drive activation of mTORC1 and inhibition of autophagy by ASFV. It was also possible that mTORC1 may have been activated independently of Akt via AMPK, which activates the TSC complex.

The possibility that mTORC1 was activated by Akt during ASFV infection was tested using MK-2206 to inhibit Akt [50, 51]. Fig. 4(b) shows cells analysed 4 h post-infection, when phosphorylation of Akt appeared to be greatest, and viral protein p30 could be clearly detected. In conventional media, raised levels of P-Akt S473 and P-Akt T308 were evident in ASFV infected cells compared to mock infected cells indicating viral activation of Akt. At the same time downstream activation of mTORC1 was indicated by phosphorylation of p70 S6K. MK-2206 blocked phosphorylation of Akt T308 in mock and virally infected cells. MK-2206 also blocked P-Akt S473 in mock infected cells but it was still possible to detect low levels of P-Akt S473 in ASFV infected cells. This suggested that MK-2206 alone can inactivate Akt in mock infected cells, but can only partially inactivate Akt in ASFV infected cells.

While the mTORC1 complex acts as a nutrient sensor upstream of autophagy, the catalytic subunit of mTORC1 (mTOR) can be incorporated into a second complex called mTORC2 that phosphorylates S473 residue of Akt independently of nutrient signalling. The preferential phosphorylation of S473 seen in infected cells suggested that ASFV may activate mTORC2. The experiments were therefore repeated for cells incubated with Torin1, which inhibits both mTORC1 and mTORC2. Torin1 resulted in a loss of P-p70-S6K and P-Akt S473 respectively (Fig. 4b). This shows that Torin1 inactivated mTORC1 and prevented downstream phosphorylation of p70-S6K, and also inhibited mTORC2-mediated phosphorylation of Akt on S473. Importantly, phosphorylation of Akt T308 was still apparent indicating activation of Akt by ASFV. This was confirmed when P-Akt T308 was lost when Akt was inhibited by MK-2206 in the presence of Torin1, or following starvation in EBSS. Collectively these results suggest that ASFV inhibits autophagy by two pathways involving Akt. Direct activation of Akt leads to phosphorylation of S473 and T308 and activation of mTORC1, and this is inhibited by MK-2206. A second pathway activates Akt indirectly through activation of mTORC2 and phosphorylation of S473.

**Fig. 5.**
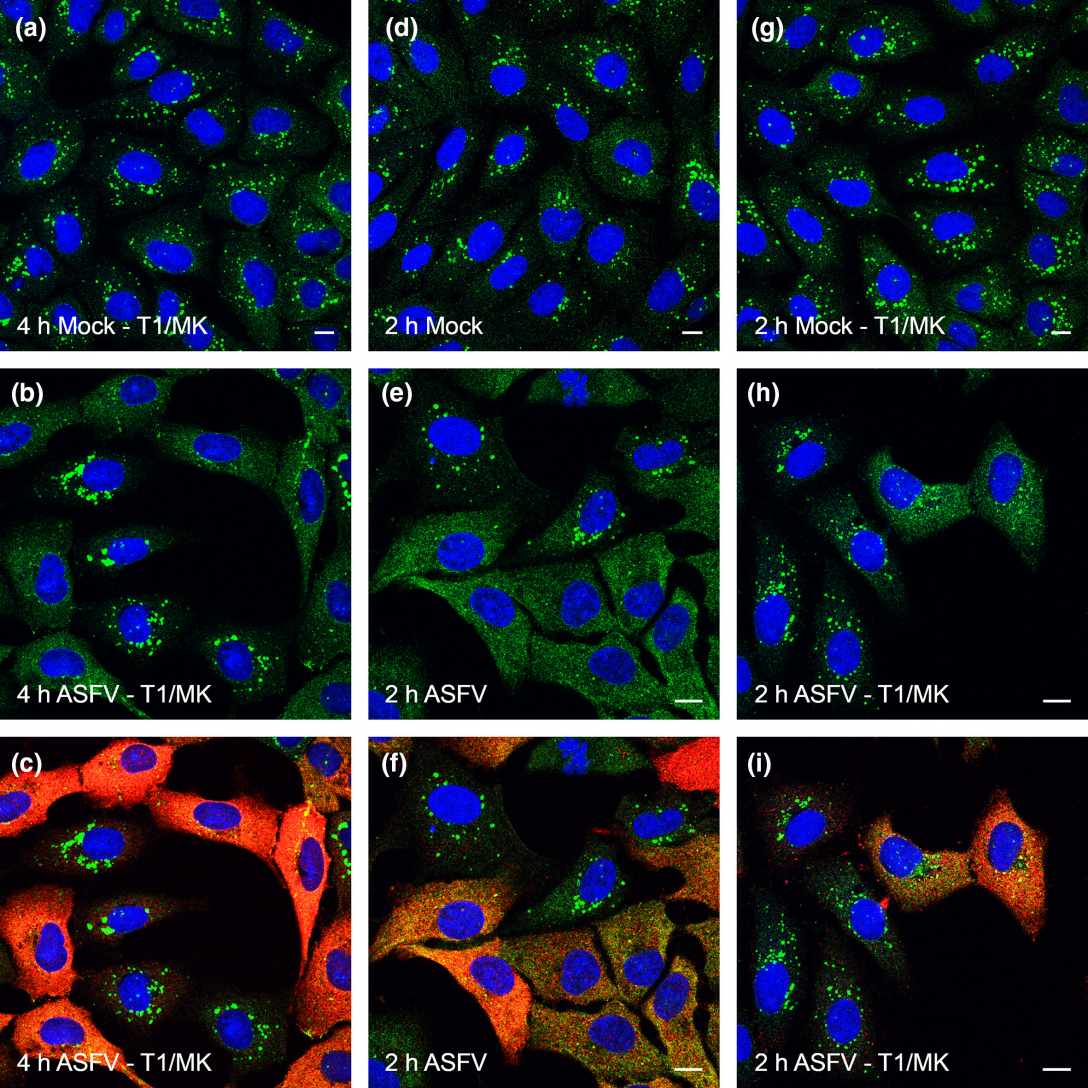
Biomodal inhibition of autophagosome formation by ASFV. Vero cells were incubated with mock inoculum (Panel a, **d, g**) or Ba71V (MOI 5) (Panels b, **c, e, f, h, i**) for 1 h. Inocula were removed and cells were incubated for a total of either 2 h (**a–f**) or 4 h (**g–i**) either in the presence (**d–i**) or absence (**a–c**) of 200 nM Torin1 and 5 µM MK-2206 (T1/MK). Cells were also starved by incubating with EBSS for the final 2 h of the incubation, i.e. throughout the 2 h incubation. Cells were then fixed and labelled with antibodies against LC3 (green), p30 (red) or with DAPI (blue). Panels c, f show the same infected cells as Panels B and E respectively but with the red channel removed to allow for clearer observation of LC3 staining. Scale bars represent 10 µm.

The effects of Torin1 and MK-2206 on generation of LC3 puncta during ASFV infection are shown in Fig. 5. Cells were preincubated with ASFV for an hour and then incubated in EBSS for 2 h to activate autophagy. Mock infected cells generated large numbers of LC3 puncta (Fig. 5a, b), which were not detected in ASFV infected cells (Fig. 5e, f). Substantial numbers of autophagosomes were however observed when infected cells were incubated in EBSS, Torin1 and MK-2206 (Fig. 5h, i). To investigate if this effect was linked to the early stages of infection the analysis was repeated 4 hpi. In contrast to 2 hpi, it was difficult to detect LC3 puncta in infected cells positive for p30 staining (Fig. 5b, c). The lower numbers of autophagosomes present in cells treated with Torin1 and MK-2206 at 4 hpi when compared to 2 hpi suggests that viral proteins expressed 4 hpi may still be able to supress induction of autophagosomes. Similar results were observed in cells infected with Ba71vΔA179L (Fig. S5) suggesting even if Akt and mTORC are inactivated A179L does not contribute to the block in autophagosome formation.

## Discussion

To initiate and sustain a productive infection, viruses require a tremendous amount of cellular resources which inevitably activates stress response pathways such as the unfolded protein response (UPR) and apoptosis. Viruses are thus required to overcome or limit the effects of these pathways and research has showed that ASFV encodes multiple modulators for this purpose [52]. Autophagy is a highly conserved process that is not only vital to the host cell stress response but also plays an integral role in both innate and adaptive immunity. Presenting such a potent threat, autophagy is specifically targeted for inhibition by numerous viruses and even harnessed by some to act in a pro-viral manner [53–56]. ASFV encodes a protein, A179L that was shown to specifically bind to the key autophagy protein Beclin-1 which forms part of the Vps34 lipid kinase complex [33]. The Vps34 complex is essential during the very early stages of the autophagy pathway and over-expression of A179L inhibits the formation of starvation-induced autophagosomes [37]. Research has showed however that viruses often modulate autophagy at multiple steps in the pathway. For example, HSV-1 inhibits Beclin-1 function but can also inhibit stimulatory signals that are situated higher up in the pathway [30, 31].

LC3-labelling for autophagosomes in Veros cells infected with the tissue culture adapted strain Ba71V showed that ASFV infected cells had reduced numbers of autophagosomes at 4 hpi when compared to control cells under nutrient replete conditions. This suggested that ASFV does not promote autophagy during the early stages of viral replication and is consistent with previous results shown at later time points during the virus replication cycle [33]. Cells were then starved to test if autophagy is inhibited by ASFV and in comparison to the high number of autophagosomes detected in control cells, a significantly lower number were detected in cells infected with ASFV, suggesting that the virus actively inhibits autophagy. Western blot analysis of LC3-II levels showed that ASFV was able to block starvation-induced accumulation of LC3-II in both cultured cells and primary macrophages at early times post-infection. Confocal experiments showed that ASFV could inhibit autophagy during later time points in Vero cells, however this would need to be confirmed in macrophages. Consistent with inhibition of autophagosome formation p62 levels in starved ASFV infected Vero cells were not significantly degraded during the early or late stages of infection suggesting that the breakdown of cargo via autophagosome-lysosome fusion, known as autophagic flux, is inhibited by ASFV. Inhibition of LC3 lipidation in ASFV infected cells is different to that observed after infection by with vaccinia virus where aberrant lipidation of LC3 is promoted by direct conjugation between ATG12 and ATG3 [57].

ASFV encodes for a Bcl2 homologue that has been shown to interact with Beclin-1 in yeast two hybrid screens and is capable of blocking autophagosome formation when overexpressed exogenously [33, 37]. However, an A179L ASFV deletion mutant virus was unable to prevent formation of autophagosomes suggesting that if A179L does play a role in inhibiting autophagy in infected cells, it is not the only means of inhibition employed by the virus. Activation of mTORC1 is often required by viruses to maintain protein translation and its activation also inhibits autophagy via the ULK1 complex. In ASFV infected Vero cells, mTORC1 was active during both early and late stages of the replication cycle and furthermore the virus was capable of blocking inactivation of mTORC1 by starvation, suggesting that mTORC1 is specifically targeted by the virus. Activation of mTORC1 was observed in a time-dependent manner, becoming increasingly apparent as infection progressed which may suggest that the expression of virally encoded proteins are required for this effect. This has been reported for HSV-1, where expression of the US3 kinase protein is directly linked to mTORC1 activation [58]. Irrespective of the underlying mechanism, the ability of ASFV to activate mTORC1 represents a clear means by which autophagy may be inhibited. The ability of ASFV to maintain mTORC1 activity under conditions of cellular stress is shared with human cytomegalovirus, highlighting the importance of this pathway for successful propagation of large double stranded DNA viruses [59, 60].

mTORC1 activation is in turn regulated by activated Akt which can be measured by analysing phosphorylation at residues S473 and T308 [49]. Akt was active throughout the replication cycle and greater levels of phosphorylation at S473 were observed in ASFV infected cells when compared to mock infected cells between 2 and 8 hpi. This suggests that mTORC and autophagy induction may be regulated at the point of Akt activation during viral infection. Phosphorylation of Akt at S473 is carried out by mTORC2 [61] which could suggest that mTORC2 activity may be stimulated by ASFV. The capacity of ASFV to inhibit autophagy induction in the absence of Akt/mTORC1 activity was investigated using pharmacological inhibitors. Both MK-2206 and Torin1 were required to block phosphorylation of Akt at both S473 and T308 in ASFV infected cells. The observation that Torin1, an mTORC2 inhibitor, was required to completely inactivate Akt in ASFV infected cells is consistent with the idea that mTORC2 may be targeted by ASFV to drive Akt activation. Techniques for the specific inhibition of mTORC2 are beginning to emerge [62] which could be useful in determining if mTORC2 plays a role in ASFV infection. Future studies could also test if Akt is exclusively responsible for the activation of mTORC1 or if ASFV may be activating mTORC1 via alternative means such as virally encoded proteins. The PI3K/Akt signalling cascade is commonly targeted by viruses that rely on cap-dependent translation such as mammalian DNA viruses as they require active mTORC1 to maintain protein translation [63, 64]. The activation of the PI3K/Akt pathway by vaccinia virus (VACV) was shown to be required for viral growth although this was also linked to the inhibition of apoptosis [65]. Similarly, flaviviruses such as dengue virus and Japanese encephalitis virus activate the PI3K/Akt pathway which has an anti-apoptotic affect, as a block in this activation induced apoptotic cell death in the early stages of infection [66]. Human papillomavirus has been shown to activate the PI3K/Akt pathway, leading to the activation of mTORC1 and the inhibition of autophagy which benefits viral replication [67].

Inhibition of autophagosome formation at 2 hpi was concomitant with activated mTORC1 and Akt, but low levels of viral gene expression, furthermore inhibition of autophagosomes at this time point could be prevented by pharmacological inhibition of Akt and mTORC1. This implies that activation of Akt was dependent on factors other than viral gene expression and it is possible that virus entry itself can induce activation of Akt as seen during VACV infection [65, 68]. As well as inhibiting autophagy via mTORC1, activated Akt can act directly on Beclin-1 [69] which could also contribute to early inhibition of autophagy after ASFV infection. By 4 hpi, early viral protein expression is well established and ASFV is able to substantially reduce the number of starvation-induced autophagosomes even in the presence of MK-2206 and Torin1. This was observed in cells infected with both wild-type virus and cells infected with Ba71vΔA179L suggesting that either A179L does not play a role in the inhibition of autophagy or that additional viral proteins are involved. ASFV particles contain 68 more viral proteins [70] that could play a role in manipulating host responses during or shortly after entry. The R298L gene encodes for a serine/threonine protein kinase that is incorporated into the virus capsid and the phosphorylation targets of this viral protein are unknown. Transcriptional mapping of the ASFV genome has identified at least thirty five genes in addition to A179L that are expressed early during ASFV replication [71] and therefore could be involved in supressing autophagosome formation.

**Fig. 6.**
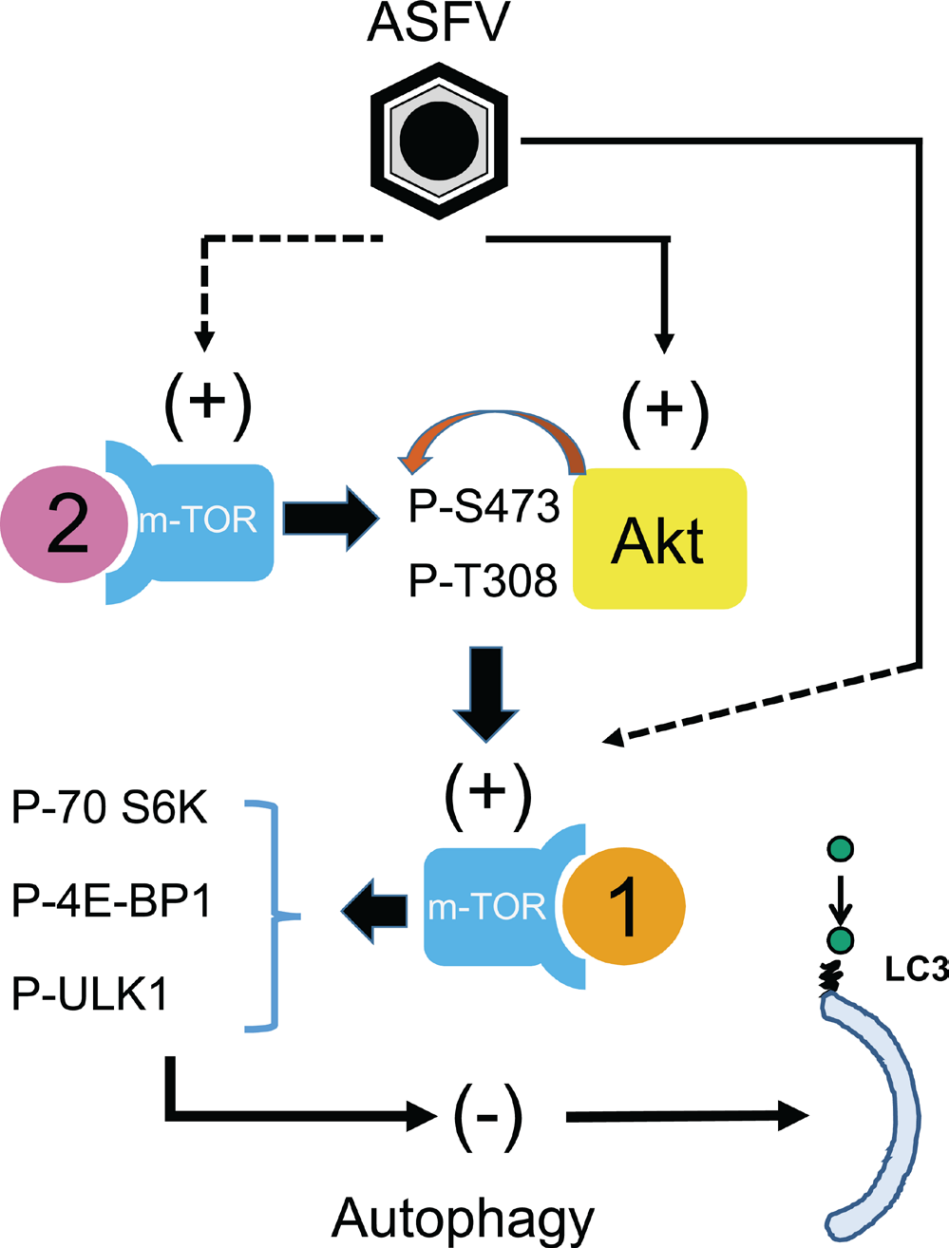
Model of ASFV autophagy modulation. ASFV activates Akt and mTORC2. Activated Akt in turn activates mTORC1 which switches off autophagy via its action on ULK1. Blocking Akt and mTORC2 activity rescues autophagy early during infection, however ASFV supresses autophagy at an unknown point later.

These studies reveal that the inhibition of autophagy by ASFV is multi-layered and linked to diverse elements of the autophagy pathway (Fig. 6). The involvement of the Akt/mTORC1 signalling cascade in addition to virally encoded modulators could point to a model of phased control. The PI3K/Akt pathway may be activated first at the point of virus entry to provide a preliminary defence mechanism against autophagy but as the viral replication cycle progresses and host cell stress responses are activated, an additional level of control is implemented by expression of viral factors. In this scenario, an attempt to limit the ability of the virus to inhibit autophagy by gene deletion would need to address both phases of modulation and consequently it may prove challenging to overcome this. Nevertheless, this work has provided further insight into the complex interaction between ASFV and the host cell. As well as implementing an immune evasion strategy, ASFV is required to optimise cell function for efficient replication as well as prolong cell survival.

## Supplementary Data

Supplementary material 1
