# Supplementary material for: Autophagy impairment by African swine fever virus
Source: J Gen Virol. 2021 Aug 18;102(8):001637. doi: 10.1099/jgv.0.001637 (PMC8513644; doi:10.1099/jgv.0.001637)
Supplement: Supplementary material 1 [file jgv-102-1637-s001.pdf]

Supplementary methods

Source and dilutions of antibodies

Name	Manufacturer	Clone/Product number	Species	Clonality	WB dilution	IF dilution
**Primary Antibodies**						
4E-BP1	Cell Signaling Technology	9452	Rabbit	Polyclonal	1:1000	
P-4E-BP1	Cell Signaling Technology	9459	Rabbit	Polyclonal	1:1000	
Akt	Cell Signaling Technology	4691	Rabbit	Monoclonal	1:1000	
P-Akt T308	Cell Signaling Technology	13038	Rabbit	Monoclonal	1:1000	
P-Akt S473	Cell Signaling Technology	4060	Rabbit	Monoclonal	1:2000	
LC3B	Sigma	L7543	Rabbit	Polyclonal		1:1000
LC3B	Cell Signaling Technology	2775	Rabbit	Polyclonal	1:1000	
p30	The Pirbright Institute	C18	Mouse	Monoclonal	1:1000	1:1000
p62	Cell Signaling Technology	88588	Mouse	Monoclonal	1:500	
p70-S6K	Cell Signaling Technology	9202	Rabbit	Polyclonal	1:1000	
P-p70-S6K	Cell Signaling Technology	9205	Rabbit	Polyclonal	1:1000	
γ-tubulin	Sigma	T6557	Mouse	Monoclonal	1:2500	
ULK1	Cell Signaling Technology	8054	Rabbit	Monoclonal	1:1000	
P-ULK1	Cell Signaling Technology	14202	Rabbit	Monoclonal	1:1000	

Primary cells and field isolates

The OUR T88/1 (Boinas, 2004) and Benin 1997/1 [Chapman, 2008) field isolates were grown and titrated by haemadsorption assay in porcine bone marrow derived macrophages. Porcine bone marrow derived macrophages were harvested from the long bones of pigs. Bones were cut into small fragments and incubated in PBS supplemented with 1% (v/v) FCS and Penicillin (100 U/ml)-Streptomycin (100 µg/ml) at 35°C for 90 minutes. The bone marrow suspension was then filtered through muslin to remove the bone fragments and centrifuged at 350 × *g* for 10 minutes. Supernatant was discarded and the cell pellet was washed in PBS. After repeating the centrifugation step, the cell pellet was resuspended in the appropriate cell medium and cells were counted and seeded into flasks. Porcine bone marrow derived macrophages were maintained in Earle's Balanced Salt Solution (EBSS; ThermoFisher Scientific) supplemented with 4 mM HEPES (ThermoFisher Scientific), 10% (v/v) porcine serum (Biosera) and Penicillin (100 U/ml)-Streptomycin (100 µg/ml).

Blood derived macrophages were prepared by sedimenting erythrocytes from whole heparinised pig blood by incubation with an equal volume of 6% dextran (Sigma) solution for 30 minutes at 37°C. White blood cells were concentrated from the supernatant by centrifugation and washed several times with PBS before plating 2 x 10^7 cells per 6-well plate in DMEM supplemented with 30% (v/v) pig serum and penicillin (100 U/ml)-streptomycin (100 µg/ml). Cells were cultured overnight to allow monocytes to adhere and then the media was changed. Two days after this cells were used in experiments.

Alveolar macrophages were collected by bronchioalveolar lung lavage, washed three times with PBS and then plated in RPMI supplemented with 10% foetal bovine serum and penicillin (100 U/ml)-streptomycin (100 µg/ml). Cells were cultured overnight and adherent cells used in experiments.

References

Boinas, F.S.; Hutchings, G.H.; Dixon, L.K.; Wilkinson, P.J. Characterization of pathogenic and non-pathogenic African swine fever virus isolates from Ornithodoros erraticus inhabiting pig premises in Portugal. J Gen Virol 2004, 85, 2177-2187, doi:10.1099/vir.0.80058-0.

Chapman, D.A.; Tcherepanov, V.; Upton, C.; Dixon, L.K. Comparison of the genome sequences of non-pathogenic and pathogenic African swine fever virus isolates. J Gen Virol 2008, 89, 397-408, doi:10.1099/vir.0.83343-0.

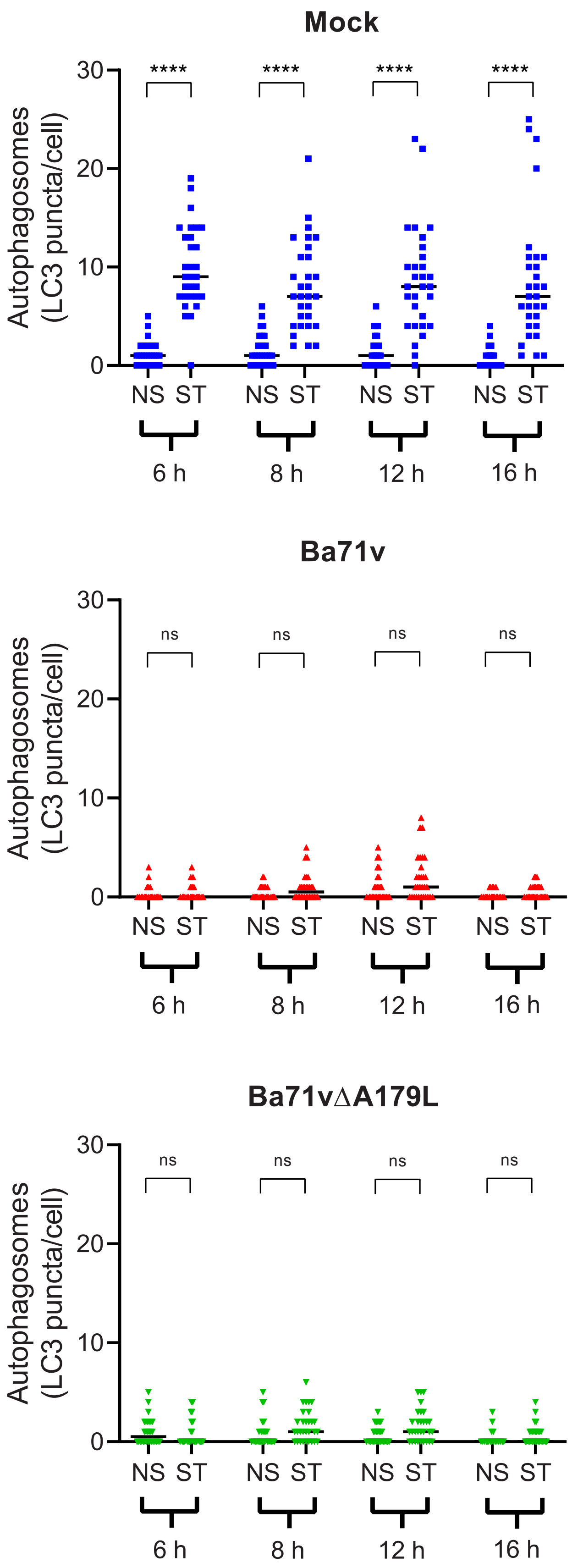

Figure S1: ASFV inhibits formation of starvation induced autophagosomes. Vero cells were incubated with mock inoculum, Ba71v or Ba71vΔA179L for 1 hour. Inocula were removed and cells were incubated for the indicated length of time during which cells were starved in EBSS for the final 2 hours. Cells were then processed for, and analysed by, confocal microscopy and the number of LC3 puncta per cell for 30 individual cells quantified by Imaris analysis (NS - complete cell media, ST - starved in EBSS). Centre lines show the medians and data was analysed using Kruskall-Wallis tests. Asterisks represent significant differences in value between NS and ST conditions (**** = P value of <0.0001).

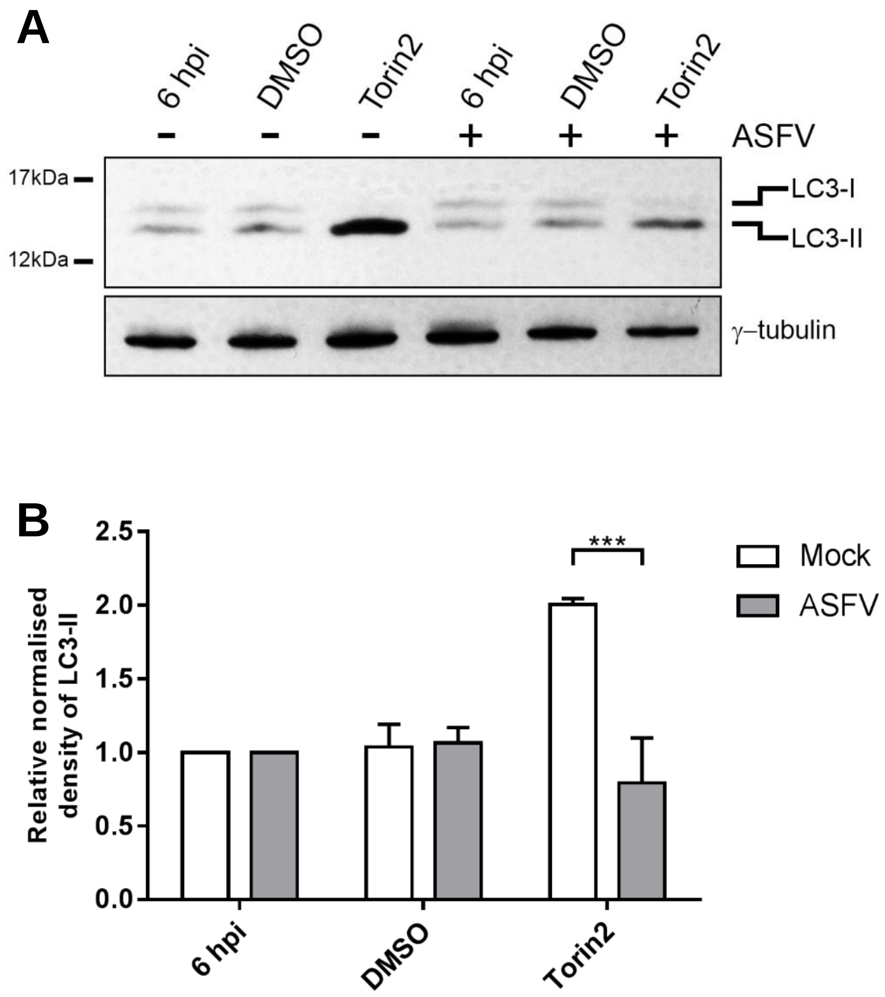

Figure S2: ASFV inhibits drug-induced accumulation of LC3-II in porcine macrophages. A. Blood derived porcine macrophages were either mock infected or infected with OUR T88/1 (MOI 5) for 6 hours (6 hpi). Separately, cells infected for a total of 8 hours were incubated for the final 2 hours in complete cell media containing DMSO or 1 μM Torin2 to induce an accumulation of LC3-II. Cells were then lysed and samples prepared for resolution by bis-Tris PAGE before transfer to PVDF membrane. Finally, samples were probed with anti-LC3 and γ-tubulin antibodies followed by appropriate HRP-conjugated secondary antibodies. The positions of molecular mass markers are indicated to the left of the gels. **B.** The relative densities of the LC3-II bands normalised to γ-tubulin in the DMSO and Torin2 treatments were calculated by comparison to those at 6 hpi. Data shown is the mean of three experiments each with cells derived from a different pig and error bars indicate SEM. Statistical analysis was carried out using one-way analysis of variance and asterisks represent a significant difference in value between mock infected and ASFV infected cells (*** = P value of <0.001).

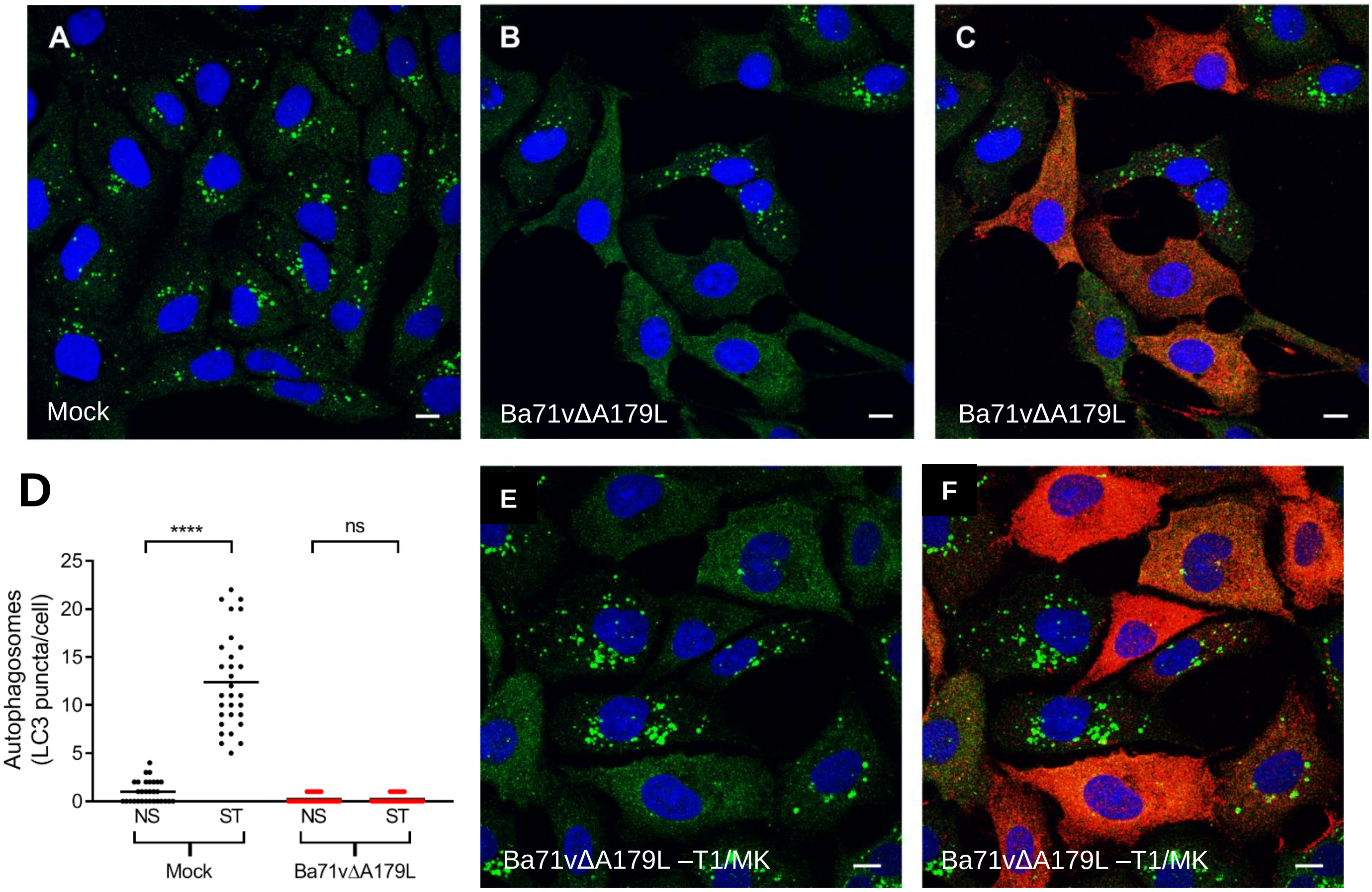

Figure S3. A179L is not required to block autophagosome formation. Vero cells were mock infected or infected with Ba71vΔA179L (MOI 5) for a total of 4 hours either in the absence (A-D) or presence (E-F) of 200 nM Torin1 and 5 µM MK-2206 (T1/MK). Cells were also incubated with media alone or EBSS for the last 2 hours. Cells were then fixed and labelled with antibodies against LC3 (green), p30 (red) and DAPI (blue) and analysed by confocal microscopy. **A-C.** Representative images of cells after starvation with EBSS. Panel B is the same as Panel C but with the red channel removed to allow for clearer observation of LC3 staining. Scale bars represent 10 µM. **D.** IMARIS analysis of LC3 puncta in mock and Ba71vΔA179L infected cells. Centre lines show the medians and statistical differences were tested by Kruskall-Wallis test. NS = Non-starved cells. ST = Starved cells. Asterisks represent significant differences in value between NS and ST conditions (*** = P value of <0.0001). **E-F.** Images of cells infected with Ba71vΔA179L and starved in the presence of 200 nM Torin1 and 5 µM MK-2206 (T1/MK).

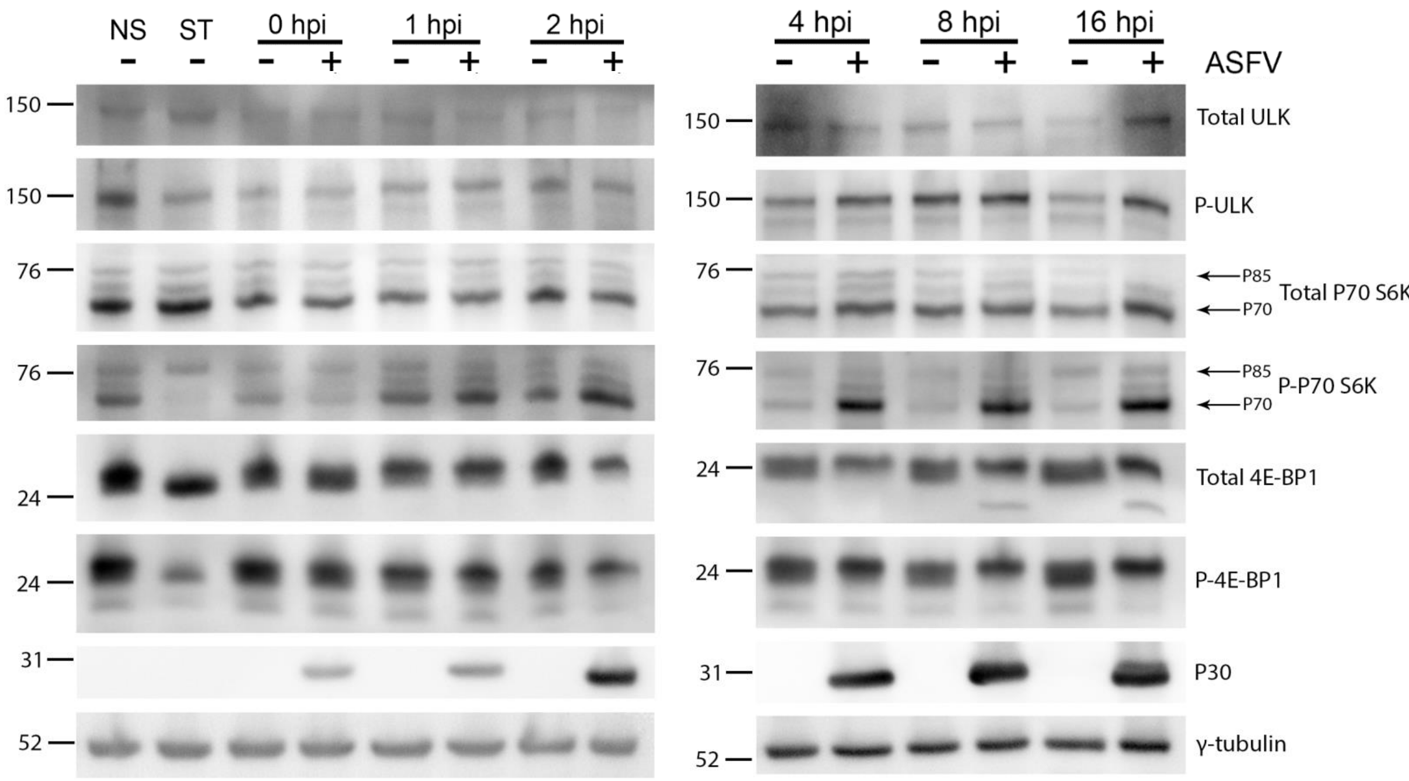

Figure S4: ASFV effect on mTORC1 in macrophages. Alveolar macrophages were either mock infected or infected with Benin for 1 hr before residual virus was washed off and the 0 hr time point was harvested. The remaining cells were incubated in media and harvested at multiple time points over a 16 hour time course of infection. Control cells were either incubated in complete cell media (NS) or starved (ST) in EBSS for 3 hours to induce inactivation of mTORC1. Cells were lysed, processed for immunoblot and probed with the indicated antibodies. The time post infection in hours (hpi) are indicated at the top of the gel, the positions of molecular mass markers are indicated to the left.

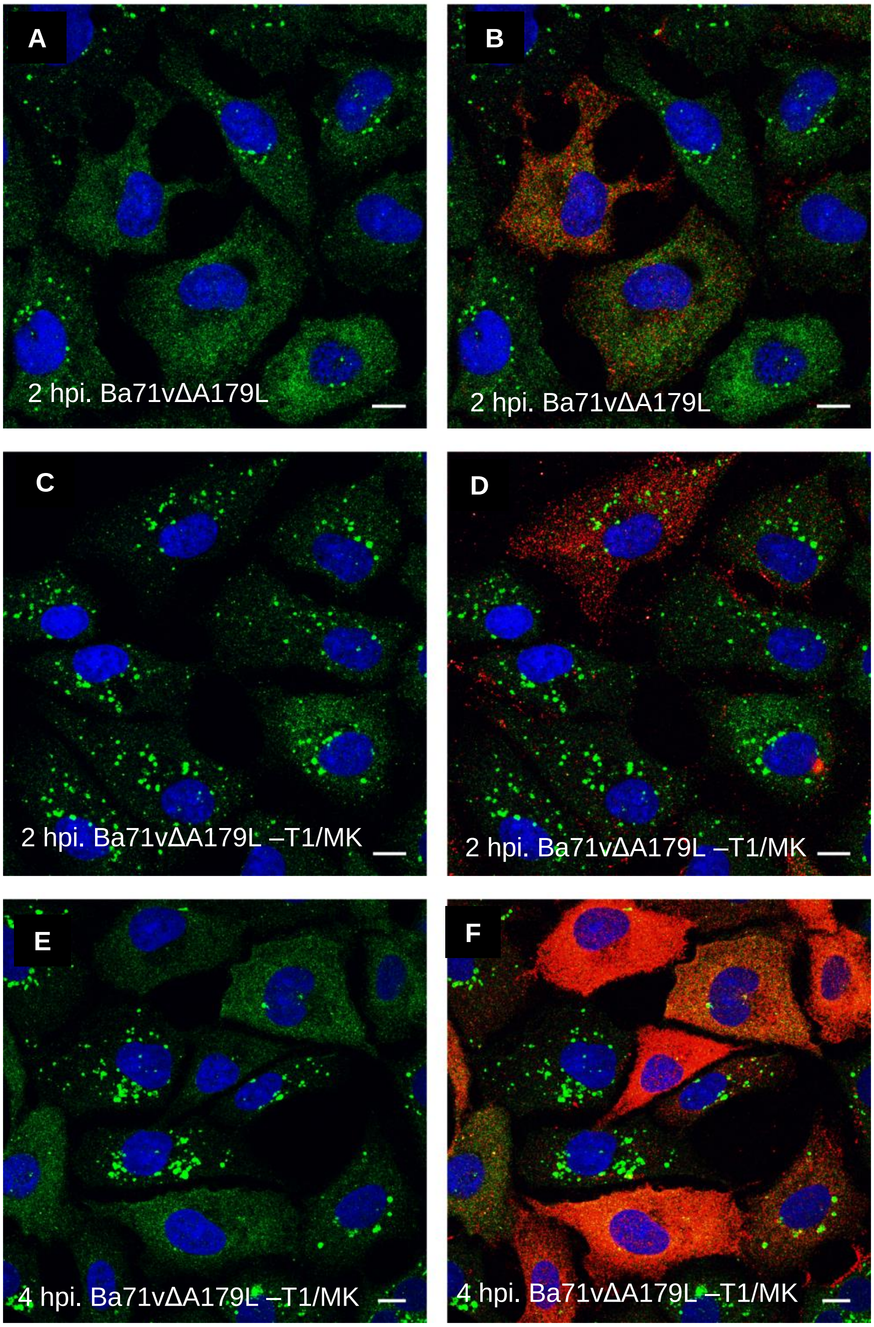

Figure S5: Early inhibition of autophagosome formation by ASFV does not require A179L. Vero cells were incubated with Ba71vΔA179L (MOI 5) for 1 hour. Inocula were removed and cells were incubated for a total of either 2 hours (A-D) or 4 hours (E,F) either in the presence (C-F) or absence (A,B) of 200 nM Torin1 and 5 µM MK-2206 (T1/MK). Cells were also starved by incubating with EBSS for the final two hours of the incubation, i.e. throughout the 2 hour incubation. Cells were then fixed and labelled with antibodies against LC3 (green), p30 (red) or with DAPI (blue). Panels B, D and F show the same infected cells as Panels A, C and E respectively but with the red channel removed to allow for clearer observation of LC3 staining. Scale bars represent 10 µM.
